# Variants in *TPO* rs2048722, *PTCSC2* rs925489 and *SEMA4G* rs4919510 affect thyroid carcinoma susceptibility risk

**DOI:** 10.1186/s12920-023-01447-5

**Published:** 2023-02-04

**Authors:** Zhen Shen, Yingjun Sun, Guohua Niu

**Affiliations:** 1 Department of Otolaryngology Head and Neck Surgery, The First Affiliated Hospital of Xi’an Jiaotong University, No. 277, Yanta West Road, Xi’an, 710000 Shaanxi China; 2 Department of Otolaryngology, Yaozhou Zone People’s Hospital, North side of the middle of Huayuan Road, Yaozhou Zone, Tongchuan, 727100 Shaanxi China

**Keywords:** Thyroid carcinoma (THCA), *TPO*, *PTCSC2*, *SEMA4G*, Susceptibility

## Abstract

**Background:**

Thyroid carcinoma (THCA) is a malignant endocrine tumor all around the world, which is influenced by genetic and environmental factors.

**Objective:**

To explore the association between *TPO* rs2048722, *PTCSC2* rs925489, *SEMA4G* rs4919510 polymorphisms and THCA susceptibility in Chinese population.

**Methods:**

We recruited 365 THCA patients and 498 normal controls for the study. Logistic regression analysis was used to evaluate the association between *TPO* rs2048722, *PTCSC2* rs925489, *SEMA4G* rs4919510 polymorphisms and THCA susceptibility. MDR was used to assess the genetic interactions among the three SNPs.

**Results:**

Overall analysis demonstrated that rs925489 of *PTCSC2* was evidently associated with increased risk of THCA in multiple genetic models (OR = 1.59, 95%CI = 1.12–2.24, *p* = 0.009). The results of stratified analysis illustrated that rs2048722 of *TPO* can significantly increase the THCA susceptibility of participants less than or equal to 44 years old and smokers. Similarly, rs925489 of *PTCSC2* obviously improved the risk of THCA among participants older than 44 years, males, smokers and drinkers. However, rs4919510 of *SEMA4G* has a protective effect on the development of THCA among participants with less than or equal to 44 years old and non-drinkers. Interestingly, there was a strong genetic interaction among the three SNPs in the occurrence of THCA risk.

**Conclusion:**

*TPO* rs2048722, *PTCSC2* rs925489 and *SEMA4G* rs4919510 polymorphisms were evidently associated with the risk of THCA in the Chinese population, which was affected by age, gender, smoking and drinking consumption.

**Supplementary Information:**

The online version contains supplementary material available at 10.1186/s12920-023-01447-5.

## Introduction

Thyroid carcinoma (THCA) is a common malignant endocrine tumor with rapid growth, accounting for 1–2% of all cancers [1]. According to the world health organization (WHO, Global cancer statistics 2018) reported in 2018, more than 576,233 new THCA patients were diagnosed, of which 41,071 appeared in death [2]. Based on the pathological characteristics of the tumor, THCA could be further divided into five types including papillary (PTC), follicular (differentiated), poorly differentiated, anaplastic and medullary [3]. Pervious epidemiology studies have reported that environmental and hereditary parameters might affect the onset of a pathology of THCA [4]. The risk of differentiated thyroid carcinoma (DTC) was observably increased among the participants older than 55 years (HR 1.78) [5]. Smoking males had an evident reduced risk of THCA [6], and the risk of THCA was lower among the recruiters who smoked and drinked at the same time (HR 0.80) [7]. Up to now, the detail of THCA molecular mechanism was still unknown, just as many other human cancers.

For the past years, an increasing evidence suggested that hereditary parameters played a crucial role in the development of THCA [8, 9]. Various susceptibility genes and single nucleotide polymorphisms (SNPs) locus of THCA were identified by genome-wide association studies (GWAS) [10, 11]. The study found that the rs2048722 CT + TT genotype of thyroid peroxidase (*TPO*) in the Japanese population had markedly higher serum anti-thyroid peroxidase antibody (*TPOAb*) levels compared with CC genotype autoimmune thyroid disease patients [12]. Another study found that rs965513 of papillary thyroid cancer susceptibility candidate gene 2 (*PTCSC2*) in the Kazakh population was apparently associated with an increased risk of PTC [13]. Furthermore, a meta-analysis, which including 12,517 cases and 15,624 controls belonged to 18 case–control researches were conducted, and the statistical analysis results confirmed that miR-608 rs4919510 polymorphism was connected with THCA susceptibility among Chinese population, and miR-608 rs4919510 targeted Semaphorin-4G (*SEMA4G*) [14]. Up to now, the relationship between *TPO* rs2048722, *PTCSC2* rs925489 and *SEMA4G* rs4919510 polymorphism and THCA sensibility and the interaction among the three SNPs in Chinese persons were not reported.

Hence, in our current research based on Chinese population, we designed a case–control study to inquire the interaction between the three SNPs (rs2048722, rs925489 and rs4919510) polymorphism and THCA risk among Chinese persons, and the interaction between the three SNPs in THCA development.

## Material and methods

### Study population

The study was approved by the ethics committee of the First Affiliated Hospital of Xi'an Jiaotong University. Meanwhile, this study was conducted in accordance with the Declaration of Helsinki. The informed documents were written by all participants prior to entering the study. Of this cohort, 365 THCA patients including 97 males and 268 females were recruited. THCA patients who was newly diagnosed by clinical factors and histopathological examination, meanwhile who has family cancer history and other diseases were excluded. In addition, a total of 498 unrelated healthy controls including 137 males and 361 females without any thyroid pathology and other cancers were recruited from the same hospital during the same time.

### DNA extraction and genotyping

In this study, 5 ml peripheral blood samples from each participants were collected by specialized technicians and then stored into test tubes containing EDTA [15]. Next, genomic DNA were isolated from blood samples following standard GoldMag whole blood genomic DNA purification kit (GoldMag Co. Ltd. Xi’an city, China) extraction procedures. DNA quality was checked utilizingNanoDrop 2000 platform (Thermo Fisher Scientific, Waltham, MA, USA). The single-nucleotide polymorphisms (SNPs) including *TPO* rs2048722, *PTCSC2* rs925489 and *SEMA4G* rs4919510 with the minor allele frequency more than 0.05 were selected from the 1000 Genomes Project (http://www.internationalgenome.org/). The corresponding amplification primers of each SNP were designed by Agena Bioscience Assay Design Suite V2.0 software (https://agenacx.com/online–tools/). The SNPs genotype were performed by MassARRAY Nanodispenser and MassARRAY iPLEX platform (both from Agena Bios 95% CIence, San Diego, CA, USA),with standard recommended instructions. Subsequently, Agena Bioscience TYPER version 4.0 software was used to manage all data, as our pervious describe [16, 17].

### Statistical analysis

The statistical analyses were conducted by SPSS 20.0 (SPSS, USA) software. Goodness-of-fit χ2 test was applied to evaluate if the selected SNPs deviated from Hardy–Weinberg equilibrium (HWE) among controls. Difference in the distribution of demographic factors and frequencies of were calculated by χ2 test among patients and controls. In addition, the risk of THCA associated with the candidate SNPs polymorphism was estimated using the odds ratio (OR) and 95% confidence interval (95% CI) after adjusting age, sex, smoking and drinking. We used MDR software (version 4.0.2) to assess the interaction of candidate SNPs for THCA. PancanQTL (http://gong-lab.hzau.edu.cn/PancanQTL/) database was applied to analyze SNPs genotype expression. In this study, all statistical tests, *p* < 0.05 was considered statistically significant.

## Results

### Characteristics of study individuals

A total of 365 THCA patients and 498 unrelated healthy controls were recruited into the current study. The demographic parameters of the study participants were shown in Table 1, the mean age was 43.98 ± 15.12 years old for THCA patients and 44.16 ± 12.37 years old for healthy controls, there was no obvious difference in age between the two groups (*p* = 0.744). Statistical analysis results showed that there were not significant difference between patients and controls in terms of sex (*p* = 0.760), smoking (*p* = 0.492), and drinking consumption (*p* = 0.638), respectively. In addition, we also made statistics on the lymph node metastasis and THCA stage of the case group. In conclusion, the cases and controls were not evidently different in terms of sex, age, smoking, and drinking consumption, thus excluding confounding factors from interfering with the study results.

**Table 1:** Characteristics of cases and controls

Variables	Cases (n = 365)	Controls (n = 498)	*p*
Age, year (mean ± SD)	43.98 ± 15.12	44.16 ± 12.37	0.744^a^
≤ 44	172 (47.10%)	236 (47.40%)	
> 44	193 (52.90%)	262 (52.60%)	
Sex			0.760^b^
Male	97 (26.60%)	137 (27.50%)	
Female	268 (73.40%)	361 (72.50%)	
Smoking			0.492^b^
Yes	161 (44.10%)	208 (41.80%)	
No	204 (55.90%)	290 (58.3%)	
Drinking			0.638^b^
Yes	170 (46.60%)	240 (48.20%)	
No	195 (53.40%)	258 (51.80%)	
Lymph node metastases			
Metastases	108 (29.60%)		
Non-metastases	257 (70.40%)		
Staging			
I, II	132 (36.20%)		
III, IV	26 (7.10%)		
Missing	207 (56.70%)		

SD: standard deviation;

*p*^a^ values were calculated from t test

*p*^b^ values were calculated from χ^2^ test

### Associations between SNPs polymorphism and THCA risk

The SNP ID, chromosome, MAF and HWE *p* value of each candidate SNP were presented in Table 2. Our results showed that the distribution of genotypes in the healthy controls was consistent with HWE (all *p* > 0.05). Multiple genetic models and allele frequencies were used to assess the relationships between the SNPs and THCA risk. Our results suggested that the variant C allele in *PTCSC2* rs925489 presented a significantly increasing THCA risk (OR = 1.51, 95% CI = 1.10- 2.07, *p* = 0.011). However, no significant difference between other SNPs (*TPO* rs2048722 and *SEMA4G* rs4919510) and TC risk were observed (*p* = 0.245 and* p* = 0.385).

**Table 2:** Basic characteristics and allele frequencies among these SNPs

SNP	Gene	Chr	Allele	MAF		HWE *p*–Value	OR (95% CI)	*p*^b^
				Case	Control			
rs2048722	*TPO*	2	A/G	0.489	0.460	0.583	1.12(0.92–1.36)	0.245
rs925489	*PTCSC2*	9	C/T	0.119	0.082	0.562	**1.51(1.10–2.07)**	0.011*
rs4919510	*SEMA4G*	10	C/G	0.447	0.468	0.368	0.92(0.76–1.11)	0.385

HWE: Hardy–Weinberg equilibrium; MAF: minor allele frequency; SNP: single nucleotide polymorphism;

*P*^*b*^ values calculated with two–sided χ2

Bold type *p^*b*^ < 0.05 indicates statistical significance

Subsequently, we evaluated the influence of *TPO* rs2048722, *PTCSC2* rs925489 and *SEMA4G* rs4919510 polymorphisms with THCA risk under four different genetic models. The results of the genetic models were listed in Table 3. In total, the polymorphism of *PTCSC2* rs925489 were observed enhancing THCA risk under the co-dominant genetic model (OR = 1.59, 95% CI = 1.12–2.24, *p* = 0.009), the dominant genetic model (OR = 1.58, 95% CI = 1.12–2.23, *p* = 0.009) and the additive model (OR = 1.54, 95% CI = 1.10–2.15, *p* = 0.010). In addition, there was no significant difference between *TPO* rs2048722 and *SEMA4G* rs4919510 polymorphisms and the risk of THCA under four genetic models (*p* > 0.05).

**Table 3:** The association between these SNPs and TC risk

SNP	Model	Genotype	Cases	Controls	OR (95%CI)	*P*
rs2048722	Co-dominant	G/G	99 (27.2%)	143 (29.7%)	1.00	
*TPO*		G/A	174 (47.8%)	233 (48.4%)	1.08(0.78–1.49)	0.636
		A/A	91 (25.0%)	105 (21.8%)	1.25(0.85–1.83)	0.254
	Dominant	G/G	99 (27.2%)	143 (29.7%)	1.00	
		G/A-A/A	265 (72.8%)	338 (70.3%)	1.13(0.84–1.53)	0.418
	Recessive	G/G-G/A	273 (75.0%)	376 (78.2%)	1.00	
		A/A	91 (25.0%)	105 (21.8%)	1.19(0.86–1.64)	0.294
	Additive	–	–	–	1.12(0.92–1.35)	0.260
rs925489	Co-dominant	T/T	280 (76.7%)	418 (83.9%)	1.00	
*PTCSC2*		T/C	83 (22.7%)	78 (15.7%)	**1.59(1.12–2.24)**	0.009**
		C/C	2 (0.60%)	2 (0.40%)	1.43(0.20–10.27)	0.721
	Dominant	T/T	280 (76.7%)	418 (83.9%)	1.00	
		T/C-C/C	85 (23.3%)	80 (16.1%)	**1.58(1.12–2.23)**	0.009**
	Recessive	T/T-T/C	363 (99.5%)	496 (99.6%)	1.00	
		C/C	2 (0.60%)	2 (0.40%)	1.31(0.18–9.41)	0.786
	Additive		–	–	**1.54(1.10–2.15)**	0.010*
rs4919510	Co-dominant	G/G	118 (32.3%)	135 (27.3%)	1.00	
*SEMA4G*		G/C	168 (46.0%)	257 (51.9%)	0.74(0.54–1.02)	0.064
		C/C	79 (21.6%)	103 (20.8%)	0.88(0.60–1.29)	0.506
	Dominant	G/G	118 (32.3%)	135 (27.3%)	1.00	
		G/C-C/C	247 (67.7%)	360 (72.7%)	0.78(0.58–1.05)	0.102
	Recessive	G/G-G/C	286 (78.4%)	392 (79.2%)	1.00	
		C/C	79 (21.6%)	103 (20.8%)	1.06(0.76–1.47)	0.786
	Additive	–	–	–	0.92(0.76–1.11)	0.385

CI, confidence interval; OR, odds ratio; SNP: single nucleotide polymorphism

Bold type *p < 0.05 indicates statistical significance

### Stratified analysis of the effect of SNPs polymorphism in demographic parameters

Furthermore, we carried out the stratification analysis to improve a more comprehensive insight into the effect of 3 SNPs (*TPO* rs2048722, *PTCSC2* rs925489, and *SEMA4G* rs4919510) in THCA. The results of statistical analysis of age, sex, smoking and drinking were shown in Table 4, Table 5, Table 6 and Table 7, respectively, and the results of lymph node stratification were shown in Additional file 1: Table S2.

**Table 4:** Relationship between these SNPs and the risk of THCA in age subgroup

Age										
SNP	Model	Genotype	> 44				≤ 44			
			Case	Control	OR (95% CI)	*P*	Case	Control	OR (95% CI)	*P*
rs2048722	Allele	G	253	187	1.00		175	272	1.00	
*TPO*		A	247	197	1.08 (0.83–1.45)	0.575	169	190	**1.38 (1.04–1.83)**	0.026*
	Co-dominant	G/G	48	66	1.00		46	80	1.00	
		G/A	91	121	1.11 (0.70–1.78)	0.653	83	112	1.19 (0.74–1.91)	0.465
		A/A	53	63	1.26 (0.74–2.15)	0.388	43	39	**1.86 (1.05–3.28)**	0.033*
	Dominant	G/G	48	66	1.00		46	80	1.00	
		G/A-A/A	144	184	1.16 (0.75–1.80)	0.498	126	151	1.37 (0.88–2.13)	0.163
	Recessive	G/G-G/A	139	187	1.00		129	192	1.00	
		A/A	53	63	1.18 (0.76–1.81)	0.459	43	39	**1.67 (1.02–2.73)**	0.041*
	Additive	–	–	–	1.12 (0.86–1.47)	0.388	–	–	**1.35 (1.02–1.79)**	0.039*
rs925489	Allele	T	336	492	1.00		307	272	1.00	
*PTCSC2*		C	50	32	**2.29 (1.44–3.64)**	< 0.001**	37	50	1.02 (0.65–1.60)	0.999
	Co-dominant	T/T	145	230	1.00		135	188	1.00	
		C/T	46	32	**2.22 (1.34–3.69)**	0.002**	37	46	1.12 (0.68–1.82)	0.662
		C/C	2	0	–	–	0	2	–	–
	Dominant	T/T	145	230	1.00		135	188	1.00	
		C/T-C/C	48	32	**2.30 (1.39–3.81)**	0.001**	37	48	1.07 (0.66–1.74)	0.780
	Recessive	T/T-C/T	191	262	1.00		172	234	1.00	
		C/C	2	0	–	–	0	2	–	–
	Additive	–	–	–	**2.32 (1.42–3.79)**	< 0.001**	–	–	1.02 (0.64–1.63)	0.936
rs4919510	Allele	G	207	277	1.00		197	250	1.00	
*SEMA4G*		C	179	243	0.99 (0.76–1.29)	0.946	147	220	0.85 (0.64–1.12)	0.255
	Co-dominant	G/G	57	77	1.00		61	58	1.00	
		C/G	93	123	1.01 (0.65–1.57)	0.962	75	134	**0.52 (0.33–0.83)**	0.006**
		C/C	43	60	0.98 (0.58–1.66)	0.935	36	43	0.80 (0.45–1.42)	0.442
	Dominant	G/G	57	77	1.00		61	58	1.00	
		C/G-C/C	136	183	1.00 (0.66–1.51)	0.998	111	177	**0.59 (0.38–0.91)**	0.017*
	Recessive	G/G-C/G	150	200	1.00		136	192	1.00	
		C/C	43	60	0.97 (0.62–1.53)	0.901	36	43	1.20 (0.73–1.97)	0.481
	Additive	–	–	–	0.99 (0.76–1.29)	0.943	–	–	0.84 (0.63–1.12)	0.241

SNP: single nucleotide polymorphism; OR: odds ratio; CI: confidence interval

*P* values were calculated by logistic regression analysis with adjusted

Bold text and *P < 0.05 or **P < 0.01 represent statistical significance

**Table 5:** Relationship between these SNPs and the risk of THCA in sex subgroup

Sex										
SNP	Model	Genotype	Male				Female			
			Case	Control	OR (95% CI)	*P*	Case	Control	OR (95% CI)	*P*
rs2048722	Allele	G	100	150	1.00		272	369	1.00	
*TPO*		A	94	122	1.16 (0.80–1.67)	0.442	262	321	1.11 (0.88–1.39)	0.377
	Co-dominant	G/G	25	41	1.00		74	102	1.00	
		G/A	50	68	1.14 (0.59–2.20)	0.687	124	165	1.06 (0.72–1.56)	0.754
		A/A	22	27	1.35 (0.61–2.97)	0.463	69	78	1.21 (0.77–1.88)	0.412
	Dominant	G/G	25	41	1.00		74	102	1.00	
		G/A-A/A	72	95	1.20 (0.65–2.23)	0.559	193	243	1.11 (0.78–1.59)	0.569
	Recessive	G/G-G/A	75	109	1.00		198	267	1.00	
		A/A	22	27	1.24 (0.63–2.43)	0.538	69	78	1.16 (0.80–1.69)	0.439
	Additive	–	–	–	1.16 (0.78–1.72)	0.464	–	–	1.10 (0.88–1.37)	0.417
rs925489	Allele	T	164	257	1.00		479	657	1.00	
*PTCSC2*		C	30	17	**2.77 (1.48–5.17)**	0.001**	57	65	1.20 (0.83–1.75)	0.334
	Co-dominant	T/T	67	121	1.00		213	297	1.00	
		C/T	30	15	**3.59 (1.74–7.41)**	< 0.001**	53	63	1.19 (0.79–1.80)	0.398
		C/C	0	1	–	–	2	1	2.48 (0.22–28.08)	0.463
	Dominant	T/T	67	121	1.00		213	297	1.00	
		C/T-C/C	30	16	**3.42 (1.67–6.98)**	< 0.001**	55	64	1.22 (0.81–1.83)	0.345
	Recessive	T/T-C/T	97	136	1.00		266	360	1.00	
		C/C	0	1	–	–	2	1	2.41 (0.21–27.20)	0.478
	Additive	–	–	–	**3.03 (1.52–6.02)**	0.001**	–	–	1.23 (0.83–1.82)	0.303
rs4919510	Allele	G	111	157	1.00		293	370	1.00	
*SEMA4G*		C	83	117	1.00 (0.69–1.46)	0.986	243	346	0.89 (0.71–1.921)	0.295
	Co-dominant	G/G	45	34	1.00		84	90	1.00	
		C/G	67	43	0.88 (0.48–1.64)	0.698	125	190	0.70 (0.48–1.03)	0.067
		C/C	25	20	1.01 (0.46–2.19)	0.981	59	78	0.83 (0.53–1.31)	0.421
	Dominant	G/G	45	34	1.00		84	90	1.00	
		C/G-C/C	92	63	0.92 (0.52–1.64)	0.778	184	268	0.74 (0.52–1.06)	0.096
	Recessive	G/G-C/G	112	77	1.00		209	280	1.00	
		C/C	25	20	1.08 (0.54–2.16)	0.821	59	78	1.04 (0.70–1.53)	0.848
	Additive	–	–	–	0.99 (0.67–1.45)	0.951	–	–	0.89 (0.71–1.12)	0.340

SNP: single nucleotide polymorphism; OR: odds ratio; CI: confidence interval

*P* values were calculated by logistic regression analysis with adjusted

Bold text and *P < 0.05 or **P < 0.01 represent statistical significance

**Table 6:** Relationship between these SNPs and the risk of THCA in smoking subgroup

Smoking										
SNP	Model	Genotype	Smoking				Non-smoking			
			Case	Control	OR (95% CI)	*P*	Case	Control	OR (95% CI)	*P*
rs2048722	Allele	G	151	230	1.00		221	289	1.00	
*TPO*		A	171	176	**1.48 (1.10–1.99)**	0.009**	185	267	0.91 (0.70–1.17)	0.451
	Co-dominant	G/G	37	71	1.00		62	72	1.00	
		G/A	77	88	1.65 (0.94–2.90)	0.082	97	145	0.73 (0.47–1.15)	0.172
		A/A	47	44	**2.14 (1.13–4.06)**	0.019*	44	61	0.84 (0.49–1.44)	0.525
	Dominant	G/G	37	71	1.00		62	72	1.00	
		G/A-A/A	124	132	**1.81 (1.07–3.06)**	0.026*	141	206	0.76 (0.50–1.16)	0.209
	Recessive	G/G-G/A	114	159	1.00		159	217	1.00	
		A/A	47	44	1.57 (0.93–2.67)	0.094	44	61	1.02 (0.65–1.62)	0.924
	Additive	–	–	–	**1.47 (1.07–2.02)**	0.019*	–	–	0.90 (0.69–1.18)	0.455
rs925489	Allele	T	283	387	1.00		360	527	1.00	
*PTCSC2*		C	39	29	**1.84 (1.11–3.05)**	0.017*	48	53	1.33 (0.88–2.00)	0.180
	Co-dominant	T/T	124	180	1.00		156	238	1.00	
		C/T	35	27	1.48 (0.80–2.74)	0.210	46	51	1.49 (0.94–2.36)	0.092
		C/C	2	1	2.48 (0.21–28.77)	0.467	1	1	–	0.109
	Dominant	T/T	124	180	1.00		156	238	1.00	
		C/T-C/C	37	28	1.52 (0.84–2.78)	0.169	48	52	1.46 (0.92–2.32)	0.105
	Recessive	T/T-C/T	159	207	1.00		204	289	1.00	
		C/C	2	1	2.33 (0.20–27.05)	0.500	0	1	–	–
	Additive	–	–	–	1.50 (0.86–2.62)	0.154	–	–	1.42 (0.90–2.23)	0.128
rs4919510	Allele	G	181	228	1.00		223	299	1.00	
*SEMA4G*		C	141	188	0.94 (0.70–1.27)	0.704	185	275	0.90 (0.70–1.16)	0.427
	Co-dominant	G/G	51	60	1.00		67	75	1.00	
		C/G	79	108	0.79 (0.47–1.35)	0.391	89	149	0.70 (0.45–1.09)	0.118
		C/C	31	40	0.77 (0.39–1.52)	0.453	48	63	0.86 (0.51–1.45)	0.567
	Dominant	G/G	51	60	1.00		67	75	1.00	
		C/G-C/C	110	148	0.79 (0.48–1.30)	0.351	137	212	0.75 (0.50–1.13)	0.171
	Recessive	G/G-C/G	130	168	1.00		156	224	1.00	
		C/C	31	40	0.89 (0.50–1.61)	0.708	48	63	1.07 (0.68–1.67)	0.775
	Additive	–	–	–	0.87 (0.62–1.21)	0.407	–	–	0.91 (0.70–1.18)	0.484

SNP: single nucleotide polymorphism; OR: odds ratio; CI: confidence interval

*P* values were calculated by logistic regression analysis with adjusted

Bold text and ^*^P < 0.05 or ^**^P < 0.01 represent statistical significance

**Table 7:** Relationship between these SNPs and the risk of THCA in drinking subgroup

Drinking										
SNP	Model	Genotype	Drinking				Non-drinking			
			Case	Control	OR (95% CI)	*P*	Case	Control	OR (95% CI)	*P*
rs2048722	Allele	G	172	254	1.00		200	265	1.00	
*TPO*		A	168	208	1.19 (0.90–1.58)	0.218	188	235	1.06 (0.81–1.38)	0.667
	Co-dominant	G/G	46	74	1.00		53	69	1.00	
		G/A	80	106	1.08 (0.66–1.78)	0.435	94	127	0.88 (0.55–1.43)	0.612
		A/A	44	51	1.39 (0.78–2.46)	0.171	47	54	1.09 (0.62–1.92)	0.765
	Dominant	G/G	46	74	1.00		53	69	1.00	
		G/A-A/A	124	157	1.18 (0.74–1.87)	0.486	141	181	0.95 (0.60–1.48)	0.807
	Recessive	G/G-G/A	126	180	1.00		147	196	1.00	
		A/A	44	51	1.32 (0.81–2.15)	0.266	47	54	1.18 (0.73–1.90)	0.501
	Additive	–	–	–	1.17 (0.88–1.56)	0.277	–	–	1.04 (0.78–1.38)	0.808
rs925489	Allele	T	303	451	1.00		340	463	1.00	
*PTCSC2*		C	37	29	**1.90 (1.14–3.15)**	0.012*	50	53	1.29 (0.85–1.94)	0.231
	Co-dominant	T/T	135	211	1.00		145	207	1.00	
		C/T	33	29	1.69 (0.96–2.99)	0.062	50	49	1.36 (0.84–2.20)	0.212
		C/C	2	0	–	–	0	2	–	–
	Dominant	T/T	135	211	1.00		145	207	1.00	
		C/T-C/C	35	29	**1.82 (1.04–3.19)**	0.036*	50	51	1.29 (0.80–2.07)	0.302
	Recessive	T/T-C/T	168	240	1.00		195	256	1.00	
		C/C	2	0	–	–	0	2	–	–
	Additive	–	–	–	**1.88 (1.10–3.21)**	0.021*	–	–	1.19 (0.75–1.90)	0.463
rs4919510	Allele	G	181	268	1.00		223	259	1.00	
*SEMA4G*		C	159	210	1.21 (0.85–1.48)	0.423	167	253	**0.77 (0.59–1.00)**	0.049*
	Co-dominant	G/G	51	74	1.00		67	61	1.00	
		C/G	79	120	0.96 (0.60–1.55)	0.875	89	137	**0.56 (0.35–0.89)**	0.014*
		C/C	40	45	1.17 (0.65–2.10)	0.598	39	58	0.60 (0.34–1.05)	0.075
	Dominant	G/G	51	74	1.00		67	61	1.00	
		C/G-C/C	119	165	1.02 (0.65–1.60)	0.927	128	195	**0.57 (0.37–0.88)**	0.012*
	Recessive	G/G-C/G	130	194	1.00		156	198	1.00	
		C/C	40	45	1.20 (0.72–1.99)	0.483	39	58	0.86 (0.53–1.40)	0.549
	Additive	–	–	–	1.07 (0.80–1.43)	0.645	–	–	**0.75 (0.56–1.00)**	0.049*

Bold text and *P* < 0.05 represent statistical significance

#### Age

Stratified results (Table 4) demonstrated that *TPO* rs2048722 was evidently increase the risk of THCA among participants less than or equal to 44 years old in multiple genetic models [allelic model: OR (95% CI) = 1.38 (1.04–1.83), *p* = 0.026; co-dominant model: OR (95% CI) = 1.86 (1.05–3.28), *p* = 0.033; recessive model: OR (95% CI) = 1.67 (1.02–2.73), *p* = 0.041; additive model: OR (95% CI) = 1.35 (1.02–1.79), *p* = 0.039]. *PTCSC2* rs925489 was significantly associated with an increased risk of THCA in people older than 44 years in the allelic model [OR (95% CI) = 2.29 (1.44–3.64), *p* < 0.001], co-dominant model [OR (95% CI) = 2.22 (1.34–3.69), *p* = 0.002], dominant model [OR (95% CI) = 2.30 (1.39–3.81), P = 0.001] and additive model [OR (95% CI) = 2.32 (1.42- 3.79), *p* < 0.001]. However, *SEMA4G* rs4919510 had a protective effect on the risk of developing THCA among participants less than or equal to 44 years old in co-dominant [OR (95% CI) = 0.52 (0.33–0.83), *p* = 0.006] and dominant model [OR (95% CI) = 0.59 (0.38–0.91), *p* = 0.017].

#### Sex

Table 5 illustrated that *PTCSC2* rs925489 was associated with increased THCA risk among males in alleles [OR (95% CI) = 2.77 (1.48–5.17), P = 0.001], co-dominance [OR (95% CI) = 3.59 (1.74–7.41), *p* < 0.001], dominant [OR (95% CI) = 3.42 (1.67–6.98), *p* < 0.001] and additive model [OR (95% CI) = 3.03 (1.52–6.02)), *p* = 0.001]. However, rs2048722 in *TPO* and rs4919510 in *SEMA4G* were not significantly associated with THCA risk in both male and female populations.

#### Smoking

Stratified results indicated (Table 6) that rs2048722 in *TPO* obviously increased susceptibility to THCA among smoking populations in multiple genetic models [allelic model: OR (95% CI) = 1.48 (1.10–1.99), *p* = 0.009; co-dominant model: OR (95% CI) = 2.14 (1.13–4.06), *p* = 0.019; dominant model: OR (95% CI) = 1.81 (1.07–3.06), *p* = 0.026; and additive model: OR (95% CI) = 1.47 (1.07–2.02), *p* = 0.019]. Rs925489 in *PTCSC2* was significantly associated with increased risk of THCA in smokers only in the allelic model [OR (95% CI) = 1.84 (1.11–3.05), *p* = 0.017]. However, *SEMA4G* rs4919510 was not found to be evidently associated with the risk of THCA in smoking stratification.

#### Drinking

Table 7 indicated that rs2048722 in *TPO* is not significantly associated with the risk of THCA in drinking stratification, while *PTCSC2* rs925489 can evidently increase the risk of THCA in drinking populations, with allele [OR (95% CI) = 1.90 (1.14–3.15), *p* = 0.012], dominant [OR (95% CI) = 1.82 (1.04–3.19), *p* = 0.036], and additive model [OR (95% CI) = 1.88 (1.10–3.21), *p* = 0.021]. Interestingly, rs4919510 in *SEMA4G* was significantly associated with reduced THCA risk among non-drinkers in multiple genetic models [allelic: OR (95% CI) = 0.77 (0.59–1.00), *p* = 0.049; co-dominant: OR (95% CI) = 0.56 (0.35–0.89), P = 0.014; dominant: OR (95% CI) = 0.57 (0.37–0.88), *p* = 0.012; and additive: OR (95% CI) = 0.75 (0.56–1.00), *p* = 0.049].

#### Lymph node metastasis

In the case group, rs2048722 in *TPO*, rs925489 in *PTCSC2* and rs4919510 in *SEMA4G* were not found to be notably correlated with lymph node metastasis.

In general, stratified analysis results demonstrated that *TPO* rs2048722 could significantly increase THCA susceptibility among participants less than or equal to 44 years old and smokers. Similarly, *PTCSC2* rs925489 evidently increased the risk of THCA in people older than 44 years, males, smokers and drinkers. However, rs4919510 in *SEMA4G* notably reduced the risk of THCA among people less than or equal to 44 years old and non-drinkers.

### Analysis of MDR

**Fig. 1:**
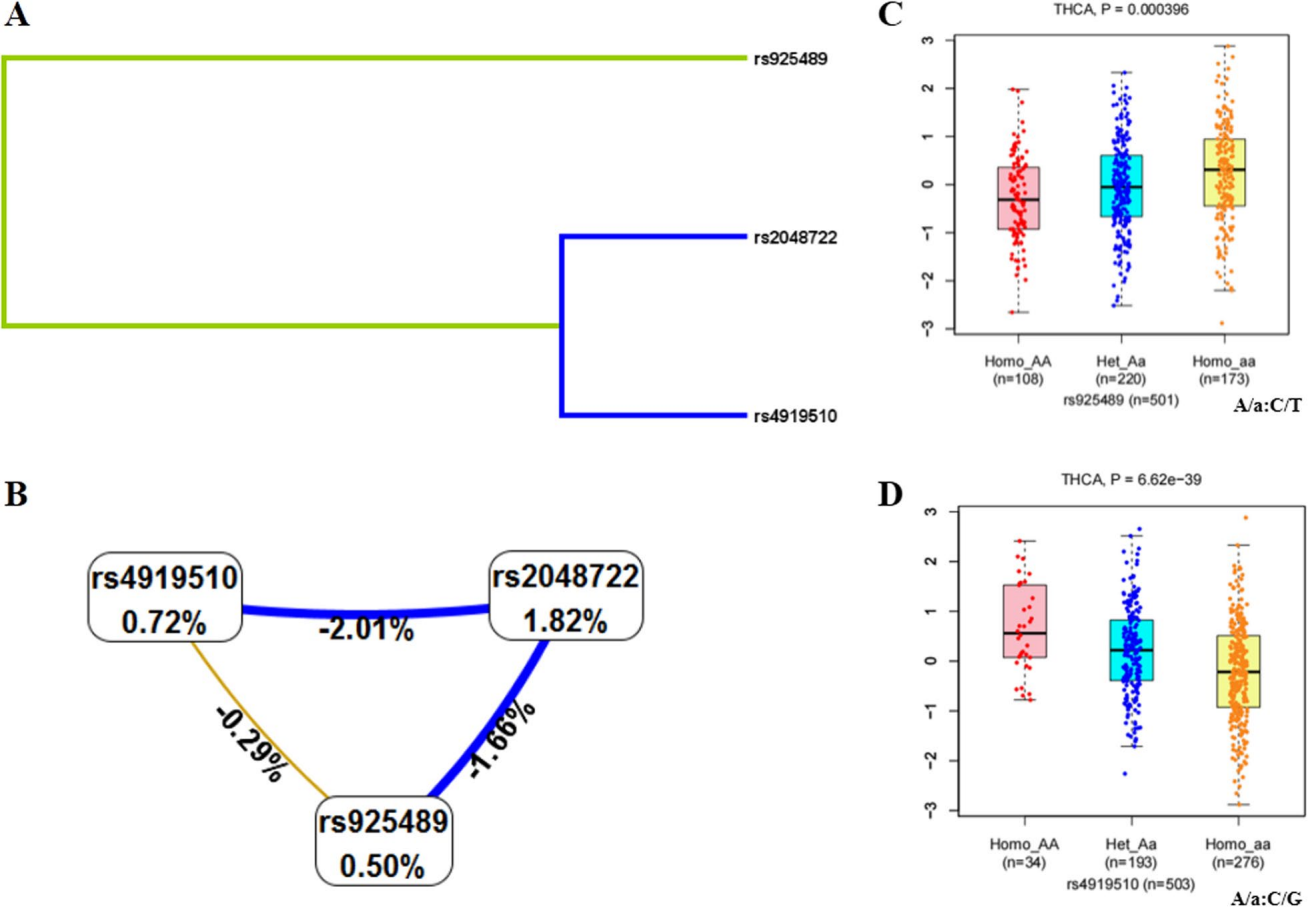
Analysis of MDR and SNP genotype expression. **A** SNP-SNP interaction dendrogram of MDR analysis. **B** Fruchterman-reingold of MDR analysis. (The closer to red the stronger the synergy, the closer to the blue the more redundancy.) **C** Rs925489 genotype expression of THCA. **D** Rs4919510 genotype expression of THCA

The MDR software was used to evaluate three SNPs with high-order interactions in THCA. Regarding the THCA risk model, the single-locus model rs925489, the two-locus model rs925489, rs4919510 and the three-locus model rs2048722, rs925489 and rs4919510 all have higher accuracy and testability, among which the three-locus model has the highest concordance of 10/10, and *p* = 0.001 (Table 8). Figure 1A and 1B indicated the interaction between the three SNPs, where the color closer to red indicates stronger synergy, and closer to blue indicates stronger redundancy. Taken together, *TOP* rs2048722, *PTCSC2* rs925489 and *SEMA4G* rs4919510 may have strong genetic interactions in the occurrence of THCA.

**Table 8:** Summary of SNP-SNP interactions on the risk of thyroid cancer analyzed by MDR method

Model	Bal.Acc.CV training	Bal.Acc.CV testing	CV consistency	OR (95% CI)	*p*
rs925489	0.536	0.484	5/10	1.513 (1.048–2.183)	0.026^*^
rs925489, rs4919510	0.557	0.501	7/10	1.577 (1.176–2.114)	0.002^*^
rs2048722, rs925489, rs4919510	0.567	0.499	10/10	1.666 (1.242–2.235)	0.001^*^

MDR: multifactor dimensionality reduction; Bal.Acc: balanced accuracy; CVC: cross-validation consistency; OR: odds ratio; 95%CI: 95% confidence interval; Bold type ^*^p < 0.05 indicates statistical significance

### Analysis of SNP genotype expression

The analysis of SNP genotype expression in THCA declared that rs925489 had significant differences among different genotypes in cis-eQTL and trans-eQTL (CC < CT < TT, Fig. 1C), indicating that the genotype change of rs925489 of THCA may directly or indirectly affect the expression of related genes. Different genotypes of rs4919510 have obvious differences in cis-eQTL (CC > CG > GG, Fig. 1D), which indicates that the genotype change of rs4919510 of THCA directly affects the expression of related genes. Unfortunately, the expression of rs2048722 different genotypes in THCA were not found.

## Discussion

As we all know, THCA is most frequent head and neck tumors, and is reported that THCA has a highly morbidity all over the world [18]. More and more researchers have given evidences that genetic factors play an important role in the pathogenesis of THCA [19]. As a membrane-bound glycoprotein, *TPO* catalyzes thyroid hormone enzymes and regulates thyroid function [20]. Various studies have been confirmed that multiple *TPO* gene mutations may give rise to dysfunction of the *TPO* enzyme and varieties human disease [21]. Aleksander et.al suggested that *TPO* rs11675434 polymorphism was related with autoimmune thyroid disease among Polish Caucasian population [22]. In addition, the study found that the rs2048722 CT + TT genotype of *TPO* had evidently higher serum anti-thyroid peroxidase antibody (*TPOAb*) levels compared with CC genotype autoimmune thyroid disease patients in the Japanese population [12]. In this study, rs2048722 in *TPO* was also found to be a significant risk gene for THCA among the Chinese population aged less than or equal to 44 years old and smoking in the stratified analysis.

As long noncoding RNAs (lncRNAs), the SNP (rs965513) in *PTCSC2* was evidently associated with PTC risk, and similar to *TPO*, *PTCSC2* also regulates thyroid hormone levels and thyroid function [23]. Similarly, *PTCSC2* is a susceptibility gene in familial non-medullary thyroid cancer [24]. Furthermore, *PTCSC2* rs965513 was obviously associated with an increased risk of PTC in the Kazakh population [13]. This study is the first to confirm that *PTCSC2* rs925489 was notably associated with increased susceptibility to THCA risk in different genetic models. Interestingly, *PTCSC2* rs925489 all evidently increased the risk of THCA in Chinese populations older than 44, males, smokers and drinkers. Taken together, genetic variation in *PTCSC2* affects the risk of developing THCA.

*SEMA4G* is known to the semaphorin family and involved over 20 genes classified into 7 difference subfamilies. It was reported that the SEMA4G gene has a DNA damage-binding and repair function [25]. The rs4919510 is located on 10q24.31 in the *SEMA4G* gene intron region. Furthermore, Wu et al. performed a meta-analysis to report that rs4919510 was significantly related with improved PTC sensibility, and rs4919510 regulated *SEMA4G* [14]. In this study, stratified analysis also demonstrated that *SEMA4G* rs4919510 was evidently associated with a reduced risk of THCA among Chinese participants less than or equal to 44 years old and non-drinkers, indicating that rs4919510 significantly reduced the risk of THCA.

Genetic variations affecting THCA susceptibility are related to age, sex, smoking and alcohol consumption. Previous studies have shown that *PCNXL2* SNPs can increase THCA risk in population older than 45 and reduce the risk of THCA among females or participants with less than or equal to 45 years old [26]. Furthermore, *IL1A* SNPs were identified as biomarkers of THCA risk in males or individuals age ≤ 48 years, while *IL1B* SNPs detected strong correlations with THCA susceptibility among women and population aged > 48 years [27]. Similar to this findings, our study revealed that *TPO* rs2048722 had higher THCA risk in participants age ≤ 44 years or smokers; *PTCSC2* rs925489 was also a risk factor for THCA susceptibility among population age > 44 years, men, smokers or drinker; and *SEMA4G* rs4919510 reduced THCA risk in recruiter age ≤ 44 years or non-drinkers. In a word, genetic variations to THCA susceptibility may be due to the involvement of age, sex, smoking, and drinking.

In this study, the association between *TPO* rs2048722, *PTCSC2* rs925489, *SEMA4G* rs4919510 polymorphisms and THCA susceptibility was explored in the Chinese population, but limitations remained. The study only studied the THCA susceptibility gene in the Chinese population, and further studies on other populations still need to be explored. In addition, it is still necessary to explore the effects of *TPO*, *PTCSC2* and *SEMA4G* expression on the biological functions and regulatory pathways related to the pathogenesis and treatment of THCA at the animal and cellular levels in the later stage of the study.

## Conclusions

In summary, by investigation of Chinese population of THCA patients and unrelated healthy controls, the association of *TPO* rs2048722, *SEMA4G* rs4919510, *PTCSC2* rs925489 polymorphism and TC susceptibility was demonstrated. Our study shown that *PTCSC2* rs925489 were observed with an increasing risk factor of THCA in the overall analysis. Stratified analysis results found that *PTCSC2* rs925489 increased the risk of THCA in the Chinese population older than 44 years, males, smokers and drinkers. *TPO* rs2048722 was an obvious risk locus of THCA in Chinese population with less than or equal to 44 years old and smokers. Nevertheless, *SEMA4G* rs4919510 was evidently associated with a reduced risk of THCA in Chinese population with less than or equal to 44 years old and non-drinkers. The purpose of this study was to find the key markers of the occurrence and treatment of THCA, in order to achieve personalized treatment.

## Supplementary Information

**Additional file 1.** SNPs primers and stratification of lymph node metastasis with THCA risk.

## Data Availability

The datasets generated and/or analysed during the current study are available in the zenodo repository (https://zenodo.org/record/6668025#.Yq_MvPkaWUk).
