# Supplementary material for: Variants in TPO rs2048722, PTCSC2 rs925489 and SEMA4G rs4919510 affect thyroid carcinoma susceptibility risk
Source: BMC Med Genomics. 2023 Feb 4;16:19. doi: 10.1186/s12920-023-01447-5 (PMC9898984; doi:10.1186/s12920-023-01447-5)
Supplement: Supplementary file 1 — Additional file 1. SNPs primers and stratification of lymph node metastasis with THCA risk. [file 12920_2023_1447_MOESM1_ESM.docx]

**Supplementary table 1 Primer sequence of these SNPs**

| SNP | 1^st^-PCRP | 2^nd^-PCRP | UEP_SEQ |
| --- | --- | --- | --- |
| rs2048722 | ACGTTGGATGTTGCCAGATGTGGAAGCACC | ACGTTGGATGGTGTGTGTCTGACTCTTGTG | caTTGTGCTCCTAGAGTCACC |
| rs925489 | ACGTTGGATGAAGAAGTCTGCCATACTGGG | ACGTTGGATGGATGTCTTAGTGGGAATGGC | agcgGTGCGGGGACTGTTAACC |
| rs4919510 | ACGTTGGATGATTCCCAAGATCCACTGGGC | ACGTTGGATGATGGAAGCTCTTGGAGATGC | TGGAGATGCCTTTTTAAACG |

**Supplementary table 2 Relationship between these SNPs and** **the risk of THCA in lymph node metastases subgroup**

| SNP | Model | Genotype | Metastases | Non-metastases | OR (95% CI) | *P* |
| --- | --- | --- | --- | --- | --- | --- |
| rs2048722 | Allele | G | 106 | 266 | 1.00 |  |
| *TPO* |  | A | 110 | 246 | 1.12 (0.82-1.54) | 0.504 |
|  | Co-dominant | G/G | 25 | 74 | 1.00 |  |
|  |  | G/A | 56 | 118 | 1.40 (0.80-2.45) | 0.236 |
|  |  | A/A | 27 | 64 | 1.26 (0.66-2.41) | 0.476 |
|  | Dominant | G/G | 25 | 74 | 1.00 |  |
|  |  | G/A-A/A | 83 | 182 | 1.35 (0.80-2.30) | 0.260 |
|  | Recessive | G/G-G/A | 81 | 192 | 1.00 |  |
|  |  | A/A | 27 | 64 | 1.01 (0.60-1.71) | 0.962 |
|  | Additive | / | / | / | 1.12 (0.82-1.54) | 0.469 |
| SNP | Model | Genotype | Metastases | Non-metastases | OR (95% CI) | *P* |
| rs925489 | Allele | T | 186 | 457 | 1.00 |  |
| *PTCSC2* |  | C | 30 | 57 | 1.29 (0.81-2.08) | 0.287 |
|  | Co-dominant | T/T | 78 | 202 | 1.00 |  |
|  |  | C/T | 30 | 53 | 1.40 (0.83-2.37) | 0.209 |
|  |  | C/C | 0 | 2 | - | - |
|  | Dominant | T/T | 78 | 202 | 1.00 |  |
|  |  | C/T-C/C | 30 | 55 | 1.35 (0.80-2.28) | 0.257 |
|  | Recessive | T/T-C/T | 108 | 255 | 1.00 |  |
|  |  | C/C | 0 | 2 | - | - |
|  | Additive | / | / | / | 1.28 (0.77-2.12) | 0.335 |
| SNP | Model | Genotype | Metastases | Non-metastases | OR (95% CI) | *P* |
| rs4919510 | Allele | G | 120 | 284 | 1.00 |  |
| *SEMA4G* |  | C | 96 | 230 | 0.99 (0.72-1.36) | 0.940 |
|  | Co-dominant | G/G | 35 | 83 | 1.00 |  |
|  |  | C/G | 50 | 118 | 1.02 (0.61-1.72) | 0.929 |
|  |  | C/C | 23 | 56 | 1.01 (0.54-1.90) | 0.976 |
|  | Dominant | G/G | 35 | 83 | 1.00 |  |
|  |  | C/G-C/C | 73 | 174 | 1.02 (0.63-1.65) | 0.938 |
|  | Recessive | G/G-C/G | 85 | 201 | 1.00 |  |
|  |  | C/C | 23 | 56 | 1.00 (0.57-1.73) | 0.988 |
|  | Additive | / | / | / | 1.01 (0.74-1.38) | 0.967 |

SNP: single nucleotide polymorphism; OR: odds ratio; CI: confidence interval.

*P* values were calculated by logistic regression analysis with adjusted.
